# Crystal structure of methyl (3*RS*,4*SR*,4a*RS*,11a*RS*,11b*SR*)-5-oxo-3,4,4a,5,7,8,9,10,11,11a-deca­hydro-3,11b-ep­oxy­azepino[2,1-*a*]iso­indole-4-carboxyl­ate

**DOI:** 10.1107/S2056989015016679

**Published:** 2015-09-12

**Authors:** Flavien A. A. Toze, Dmitry S. Poplevin, Fedor I. Zubkov, Eugeniya V. Nikitina, Ciara Porras, Victor N. Khrustalev

**Affiliations:** a Department of Chemistry, University of Douala, Faculty of Sciences, PO Box 24157, Douala, Republic of Cameroon; b Department of Organic Chemistry, Peoples’ Friendship University of Russia, 6 Miklukho-Maklay St, Moscow 117198, Russian Federation; c Department of Chemistry & Biology, New Mexico Highlands University, 803 University Ave, Las Vegas, NM 87701, USA; d Department of Inorganic Chemistry, Peoples’ Friendship University of Russia, 6 Miklukho-Maklay St, Moscow 117198, Russian Federation

**Keywords:** crystal structure, 3,6a-ep­oxy­iso­indoles, azepane, intra­molecular cyclo­addition, C—H⋯O hydrogen bonds

## Abstract

The title compound, C_15_H_19_NO_4_, is the a product of the esterification of the corresponding carbonic acid with methanol. The mol­ecule comprises a fused tetra­cyclic system containing three five-membered rings (2-pyrrolidinone, tetra­hydro­furan and di­hydro­furan) and one seven-membered ring (azepane). The five-membered rings have the usual envelope conformations, with the quaternary C atom being the flap atom for the 2-pyrrolidinone ring, and the ether O atom being the common flap atom for the remaining rings. The seven-membered azepane ring adopts a chair conformation with the methine and middle methyl­ene C atoms lying above and below the mean plane defined by the remaining five atoms. The carboxyl­ate substituent is rotated by 77.56 (5)° with respect to the base plane of the tetra­hydro­furan ring. In the crystal, the mol­ecules are bound by weak C—H⋯O hydrogen-bonding inter­actions into puckered layers parallel to (001).

## Related literature   

For the synthesis of 2-(furan-2-yl)azepane, see: Asher *et al.* (1981 ▸); Shono *et al.* (1981 ▸); Nikolic & Beak (1997 ▸). For intra­molecular cyclo­addition reactions of α,β-unsaturated acid anhydrides to α-furyl­amines (IMDAF reactions), see: Vogel *et al.* (1999 ▸); Zubkov *et al.* (2005 ▸). For related compounds, see: Zylber *et al.* (1995 ▸); Evans *et al.* (1999 ▸); Kachkovskyi & Kolodiazhnyi (2007 ▸); Kharitonov *et al.* (2009 ▸); Aabid *et al.* (2010 ▸); Zubkov *et al.* (2010 ▸, 2011 ▸, 2014 ▸); Toze *et al.* (2011 ▸); Wang & Li (2012 ▸); Zaytsev, Mikhailova *et al.* (2012 ▸); Zaytsev, Zubkov *et al.* 2012 ▸); Zaytsev *et al.* (2013 ▸); Chen *et al.* (2013 ▸); Hizartzidis *et al.* (2014 ▸).
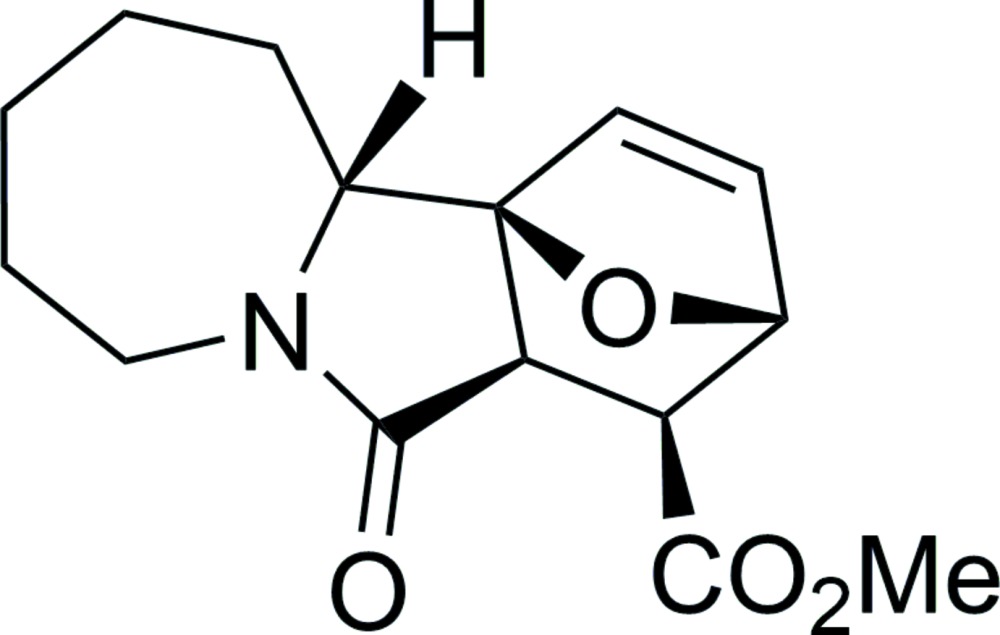


## Experimental   

### Crystal data  

C_15_H_19_NO_4_
*M* *_r_* = 277.31
Triclinic, 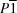

*a* = 7.5460 (8) Å
*b* = 9.6984 (10) Å
*c* = 10.2894 (10) Å
α = 103.857 (2)°
β = 94.745 (2)°
γ = 106.620 (2)°
*V* = 691.24 (12) Å^3^
*Z* = 2
Mo *K*α radiation
μ = 0.10 mm^−1^
*T* = 290 K
0.30 × 0.25 × 0.25 mm

### Data collection  

Bruker APEXII CCD diffractometer
Absorption correction: multi-scan (*SADABS*; Bruker, 2003 ▸) *T*_min_ = 0.959, *T*_max_ = 0.969
9699 measured reflections
3268 independent reflections
2613 reflections with *I* > 2σ(*I*)
*R*_int_ = 0.018

### Refinement  

*R*[*F* ^2^ > 2σ(*F* ^2^)] = 0.048
*wR*(*F* ^2^) = 0.141
*S* = 1.03
3268 reflections
182 parameters
H-atom parameters constrained
Δρ_max_ = 0.37 e Å^−3^
Δρ_min_ = −0.24 e Å^−3^

### 

Data collection: *APEX2* (Bruker, 2005 ▸); cell refinement: *SAINT* (Bruker, 2001 ▸); data reduction: *SAINT*; program(s) used to solve structure: *SHELXS97* (Sheldrick, 2008 ▸); program(s) used to refine structure: *SHELXL2014* (Sheldrick, 2015 ▸); molecular graphics: *SHELXTL* (Sheldrick, 2008 ▸); software used to prepare material for publication: *SHELXTL*.

## Supplementary Material

Crystal structure: contains datablock(s) global, I. DOI: 10.1107/S2056989015016679/tk5384sup1.cif

Structure factors: contains datablock(s) I. DOI: 10.1107/S2056989015016679/tk5384Isup2.hkl

**Fig. 1.**
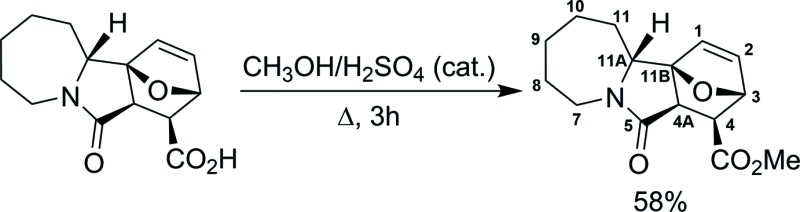
Esterification of 5-oxo-3,4,4a,5,7,8,9,10,11,11a-decahydro-3,11b-epoxyazepino[2,1-a]isoindole-4-carboxylic acid with methanol.

Supporting information file. DOI: 10.1107/S2056989015016679/tk5384Isup3.cml

a b a . DOI: 10.1107/S2056989015016679/tk5384fig1.tif
Esterification of 5-oxo-3,4,4a,5,7,8,9,10,11,11*a*-deca­hydro-3,11*b*-ep­oxy­azepino[2,1-*a*]iso­indole-4-carb­oxy­lic acid with methanol.

**Fig. 2.**
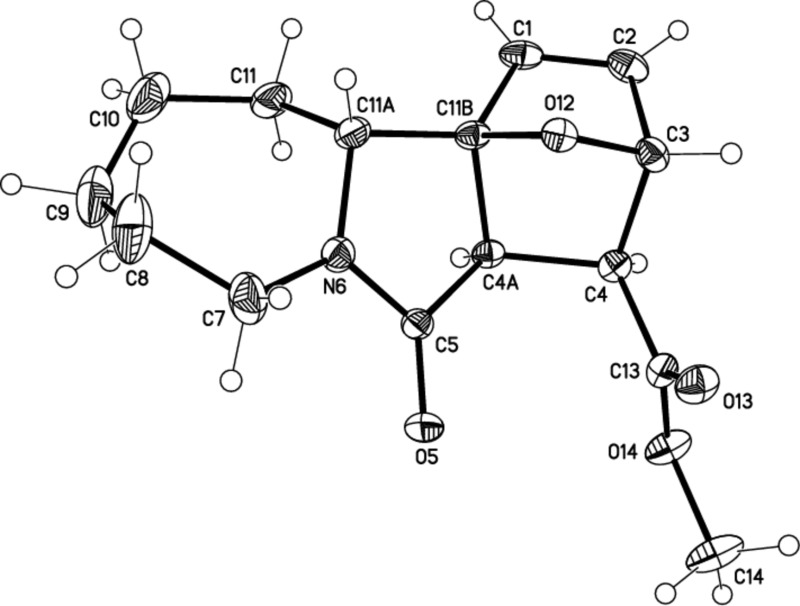
Molecular structure of (I). Displacement ellipsoids are shown at the 50% probability level. H atoms are presented as small spheres of arbitrary radius.

**Fig. 3.**
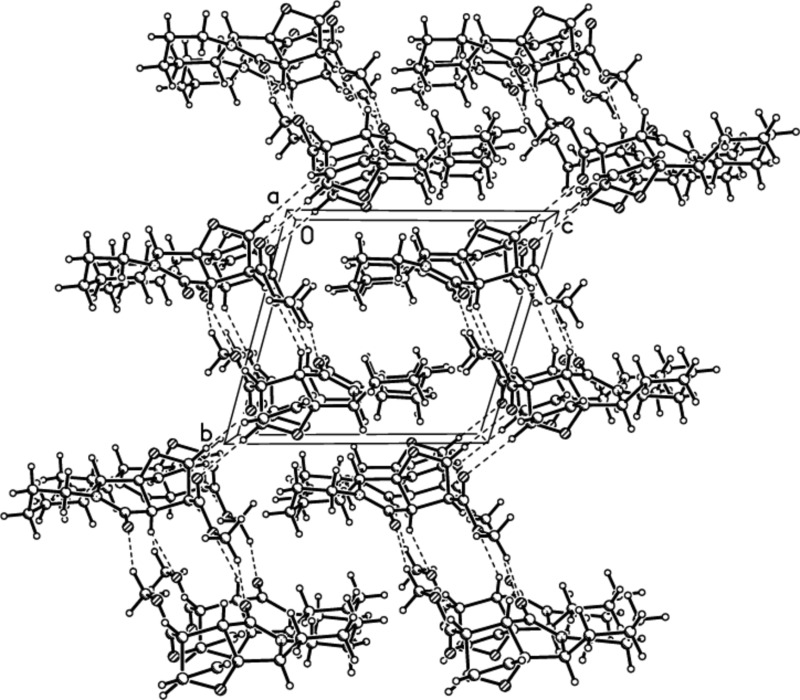
Crystal packing of (I) along the a axis demonstrating the H-bonded puckered layers parallel to (001). Dashed lines indicate the weak intermolecular C—H···O hydrogen-bonding interactions.

. DOI: 10.1107/S2056989015016679/tk5384fig2.tif
Mol­ecular structure of (I). Displacement ellipsoids are shown at the 50% probability level. H atoms are presented as small spheres of arbitrary radius.

a . DOI: 10.1107/S2056989015016679/tk5384fig3.tif
Crystal packing of (I) along the *a* axis demonstrating the H-bonded puckered layers parallel to (001). Dashed lines indicate the weak inter­molecular C—H⋯O hydrogen-bonding inter­actions.

CCDC reference: 1422681

Additional supporting information:  crystallographic information; 3D view; checkCIF report

## Figures and Tables

**Table 1:** Hydrogen-bond geometry (, )

*D*H*A*	*D*H	H*A*	*D* *A*	*D*H*A*
C1H1O5^i^	0.93	2.59	3.4576(19)	156
C3H3O13^ii^	0.98	2.55	3.5259(19)	174
C4*A*H4*A*O14^iii^	0.98	2.51	3.4190(17)	154
C14H14*A*O5^iv^	0.96	2.56	3.279(2)	132

Symmetry codes: (i) 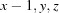; (ii) 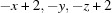; (iii) 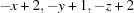; (iv) 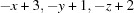.

## supplementary crystallographic information

### S1. Structural commentary

In the last decade our synthetic group has published some effective strategies for the synthesis of 3,6a-ep­oxy­iso­indoles annulated with various heterocycles (Zubkov *et al.* 2010, 2011, 2014; Zaytsev, Mikhailova *et al.* 2012; Zaytsev, Zubkov *et al.* 2012; Zaytsev *et al.* 2013). These strategies were based on the intra­molecular cyclo­addition reaction of α,β-unsaturated acid anhydrides to the heterocycles containing an α-furfuryl­amine fragment (IMDAF reaction) (Vogel *et al.*, 1999; Zubkov *et al.*, 2005). Therefore, within the scope of this investigation, the initial carb­oxy­lic acid was easily synthesized by the treatment of 2-(2-furyl)perhydro­azepine (Asher *et al.*, 1981; Shono *et al.*, 1981; Nikolic *et al.*, 1997) with maleic anhydride.

This work reports the structural characterization of 3,6a-ep­oxy­iso­indole annulated with perhydro­azepine ring. The esterification of the initial carb­oxy­lic acid obtained as fine-crystalline powder is due to its poor solubility in most common organic solvents (Fig. 1).

The molecule of the title compound, C_15_H_19_NO_4_, (I) comprises a fused tetra­cyclic system containing three five-membered rings (2-pyrrolidinone, tetra­hydro­furan and di­hydro­furan) and one seven-membered ring (1,4-diazepine) (Fig. 2). The 2-pyrrolidinone, tetra­hydro­furan and di­hydro­furan five-membered rings have the usual envelope conformations (Zylber *et al.*, 1995; Evans *et al.*, 1999; Kachkovskyi *et al.*, 2007; Kharitonov *et al.*, 2009; Aabid *et al.*, 2010; Toze *et al.*, 2011; Wang *et al.*, 2012; Chen *et al.*, 2013; Hizartzidis *et al.*, 2014). The seven-membered diazepine ring adopts a chair conformation. The nitro­gen N6 atom has the slightly pyramidalized geometry (sum of the bond angles is 359.7 (4)°). The dihedral angle between the basal plane of the diazepine ring (C8—C9/C11—C11A) and the base plane of the pyrrolidinone ring (C4—C5—N6—C11A) is 66.55 (9)°). The carboxyl­ate substituent is rotated by 77.56 (5)° to the base plane of the tetra­hydro­furan ring.

The molecule of (I) possesses five asymmetric centers at the C3, C4, C4A, C11A and C11B carbon atoms and can have potentially numerous diastereomers. The crystal of (I) is racemic and consists of enanti­omeric pairs with the following relative configuration of the centers: *rac*-3*R**,4*S**,4A*R**,11A*R**,11B*S**.

In the crystal, the molecules of (I) are bound by the weak inter­molecular C—H···O hydrogen bonding inter­actions into puckered layers parallel to (001) (Fig. 3, Table 1).

### S2. Synthesis and crystallization

A solution of the initial acid – (3*R**,4*S**,4a*R**,11a*R**,11b*S**)-5-oxo-3,4,4a,5,7,8,9,10,11,11a-deca­hydro-3,11b-ep­oxy­azepino[2,1-*a*]iso­indole-4-carb­oxy­lic acid (0.5 g, 1.8 mmol) in methanol (30 mL) was refluxed for 3 h in the presence of catalytic amount of concentrated H_2_SO_4_ (monitoring by TLC until disappearance of the starting compound (eluent – EtOAc:hexane (1:3), Sorbfil). Then the solvent was evaporated. The residual oil was passed through a thin layer of aluminum oxide (eluent – chloro­form). Chloro­form was removed under reduced pressure. The crude ester was recrystallized from a mixture of EtOAc-EtOH to give the target compound I as white crystals. Yield is 0.31 g (58%). Single-crystals were isolated as fine needles by slow re-crystallization from EtOAc-EtOH. M.pt = 388–389 K. IR (KBr), ν/cm^-1^: 1741 (C=O), 1693 (C=O). Mass spectrum, EI–MS (70 eV), *m/z* (I_r_(%)): 230 (5), 189 (52), 146 (96), 118 (19), 96 (100), 91 (17), 77 (18), 70 (17), 44 (33), 42 (25), 41 (18). ^1^H NMR (CDCl_3_, 400 MHz, 300 K): δ = 1.64–1.25 (m, 3H, H9B, H10A, H10B), 2.00–1.92 (m, 4H, H11B, H8A, H8B, H9A), 2.25–2.22 (m, 1H, H11A), 2.71 (d, 1H, H4, *J*_4,4a_ = 9.0), 2.86 (d, 1H, H4a, *J*_4a,4_ = 9.0), 3.18–3.14 (m, 1H, H7B), 3.78–3.73 (m, 1H, H11A), 3.79 (s, 3H, CO_2_Me), 3.96 (m, 1H, H7A), 5.15 (d, 1H, H3, *J*_3,2_ = 1.7), 6.47 (dd, 1H, H2, *J*_2,1_ = 6.5, *J*_2,3_ = 1.7), 6.53 (d, 1H, H1, *J*_1,2_ = 6.5). ^13^C NMR (CDCl_3_, 100 MHz, 300 K): δ = 26.6, 27.6, 29.2, 33.6 (C8, C9, C10, C11), 43.5, 45.4, 49.6, 52.1 (C7, C4A, C13, C4), 59.6 (C11A), 81.2 (C3), 92.1 (C11B), 135.2, 137.4 (C1, C2), 170.5, 172.5 (CO_2_Me, NCO).

### S3. Refinement

The hydrogen atoms were placed in calculated positions with C—H = 0.93–0.98 Å and refined in the riding model with fixed isotropic displacement parameters [*U*_iso_(H) = 1.5*U*_eq_(C) for CH_3_ and *U*_iso_(H) = 1.2*U*_eq_(C) for remaining H].

### Crystal data

**Table:**

C_15_H_19_NO_4_	*Z* = 2
*M**_r_* = 277.31	*F*(000) = 296
Triclinic, *P*1	*D*_x_ = 1.332 Mg m^−^^3^
*a* = 7.5460 (8) Å	Mo *K*α radiation, λ = 0.71073 Å
*b* = 9.6984 (10) Å	Cell parameters from 4971 reflections
*c* = 10.2894 (10) Å	θ = 2.3–29.5°
α = 103.857 (2)°	µ = 0.10 mm^−^^1^
β = 94.745 (2)°	*T* = 290 K
γ = 106.620 (2)°	Prism, colourless
*V* = 691.24 (12) Å^3^	0.30 × 0.25 × 0.25 mm

### Data collection

**Table:**

Bruker APEXII CCD diffractometer	2613 reflections with *I* > 2σ(*I*)
Radiation source: fine-focus sealed tube	*R*_int_ = 0.018
φ and ω scans	θ_max_ = 28.0°, θ_min_ = 2.1°
Absorption correction: multi-scan (*SADABS*; Bruker, 2003)	*h* = −9→9
*T*_min_ = 0.959, *T*_max_ = 0.969	*k* = −12→12
9699 measured reflections	*l* = −13→13
3268 independent reflections	

### Refinement

**Table:**

Refinement on *F*^2^	Primary atom site location: difference Fourier map
Least-squares matrix: full	Secondary atom site location: difference Fourier map
*R*[*F*^2^ > 2σ(*F*^2^)] = 0.048	Hydrogen site location: inferred from neighbouring sites
*wR*(*F*^2^) = 0.141	H-atom parameters constrained
*S* = 1.03	*w* = 1/[σ^2^(*F*_o_^2^) + (0.0765*P*)^2^ + 0.153*P*] where *P* = (*F*_o_^2^ + 2*F*_c_^2^)/3
3268 reflections	(Δ/σ)_max_ < 0.001
182 parameters	Δρ_max_ = 0.37 e Å^−^^3^
0 restraints	Δρ_min_ = −0.24 e Å^−^^3^

### Special details

**Table:**

Geometry. All e.s.d.'s (except the e.s.d. in the dihedral angle between two l.s. planes) are estimated using the full covariance matrix. The cell e.s.d.'s are taken into account individually in the estimation of e.s.d.'s in distances, angles and torsion angles; correlations between e.s.d.'s in cell parameters are only used when they are defined by crystal symmetry. An approximate (isotropic) treatment of cell e.s.d.'s is used for estimating e.s.d.'s involving l.s. planes.

### Fractional atomic coordinates and isotropic or equivalent isotropic displacement parameters (Å^2^)

**Table:**

	*x*	*y*	*z*	*U*_iso_*/*U*_eq_	
C1	0.55178 (19)	0.17386 (17)	0.75675 (18)	0.0471 (4)	
H1	0.4515	0.2009	0.7244	0.056*	
C2	0.5710 (2)	0.12672 (19)	0.86511 (18)	0.0491 (4)	
H2	0.4866	0.1136	0.9255	0.059*	
C3	0.7587 (2)	0.09790 (16)	0.87191 (15)	0.0403 (3)	
H3	0.7723	0.0281	0.9240	0.048*	
C4	0.91211 (19)	0.25575 (15)	0.91807 (14)	0.0356 (3)	
H4	0.8735	0.3221	0.9899	0.043*	
C4A	0.89714 (17)	0.30474 (14)	0.78642 (13)	0.0320 (3)	
H4A	0.8720	0.4006	0.8028	0.038*	
C5	1.04722 (18)	0.29945 (15)	0.69682 (14)	0.0352 (3)	
O5	1.21692 (14)	0.35060 (14)	0.73347 (12)	0.0513 (3)	
N6	0.96070 (17)	0.23177 (15)	0.56738 (12)	0.0414 (3)	
C7	1.0663 (3)	0.2066 (3)	0.45696 (19)	0.0663 (5)	
H7A	1.0931	0.1134	0.4506	0.080*	
H7B	1.1852	0.2864	0.4788	0.080*	
C8	0.9697 (5)	0.1996 (3)	0.3221 (2)	0.0920 (8)	
H8A	0.8776	0.1011	0.2861	0.110*	
H8B	1.0616	0.2089	0.2615	0.110*	
C9	0.8730 (4)	0.3136 (3)	0.3181 (2)	0.0914 (8)	
H9A	0.9526	0.4088	0.3778	0.110*	
H9B	0.8637	0.3246	0.2268	0.110*	
C10	0.6832 (4)	0.2847 (3)	0.3564 (2)	0.0873 (8)	
H10A	0.6030	0.1913	0.2941	0.105*	
H10B	0.6353	0.3632	0.3411	0.105*	
C11	0.6606 (3)	0.2749 (2)	0.4981 (2)	0.0620 (5)	
H11A	0.5277	0.2389	0.5017	0.074*	
H11B	0.7103	0.3751	0.5592	0.074*	
C11A	0.7550 (2)	0.17431 (17)	0.55118 (15)	0.0423 (3)	
H11C	0.7081	0.0719	0.4914	0.051*	
C11B	0.72833 (18)	0.17475 (15)	0.69564 (14)	0.0353 (3)	
O12	0.76978 (13)	0.05080 (10)	0.73010 (10)	0.0362 (2)	
C13	1.1022 (2)	0.24951 (16)	0.96810 (14)	0.0369 (3)	
O13	1.15577 (16)	0.14282 (13)	0.93999 (12)	0.0503 (3)	
O14	1.20359 (16)	0.38062 (13)	1.05547 (12)	0.0547 (3)	
C14	1.3882 (3)	0.3891 (3)	1.1134 (2)	0.0785 (7)	
H14A	1.4432	0.4823	1.1823	0.118*	
H14B	1.4649	0.3831	1.0437	0.118*	
H14C	1.3793	0.3076	1.1528	0.118*	

### Atomic displacement parameters (Å^2^)

**Table:**

	*U*^11^	*U*^22^	*U*^33^	*U*^12^	*U*^13^	*U*^23^
C1	0.0251 (7)	0.0436 (8)	0.0698 (11)	0.0131 (6)	0.0051 (7)	0.0090 (7)
C2	0.0352 (8)	0.0505 (9)	0.0634 (10)	0.0136 (6)	0.0208 (7)	0.0147 (7)
C3	0.0389 (7)	0.0414 (8)	0.0462 (8)	0.0148 (6)	0.0143 (6)	0.0171 (6)
C4	0.0346 (7)	0.0389 (7)	0.0350 (7)	0.0170 (6)	0.0074 (5)	0.0063 (5)
C4A	0.0276 (6)	0.0300 (6)	0.0393 (7)	0.0131 (5)	0.0028 (5)	0.0074 (5)
C5	0.0299 (6)	0.0378 (7)	0.0428 (7)	0.0134 (5)	0.0052 (5)	0.0169 (6)
O5	0.0271 (5)	0.0681 (8)	0.0603 (7)	0.0106 (5)	0.0047 (5)	0.0266 (6)
N6	0.0388 (7)	0.0508 (7)	0.0387 (6)	0.0172 (6)	0.0076 (5)	0.0158 (5)
C7	0.0728 (13)	0.0911 (15)	0.0516 (10)	0.0372 (11)	0.0287 (9)	0.0299 (10)
C8	0.153 (3)	0.0921 (17)	0.0439 (11)	0.0550 (17)	0.0284 (13)	0.0186 (11)
C9	0.135 (3)	0.0910 (17)	0.0566 (12)	0.0335 (16)	0.0119 (14)	0.0391 (12)
C10	0.105 (2)	0.0797 (15)	0.0733 (14)	0.0216 (14)	−0.0235 (14)	0.0358 (12)
C11	0.0454 (9)	0.0690 (12)	0.0734 (12)	0.0160 (8)	−0.0107 (8)	0.0323 (10)
C11A	0.0372 (7)	0.0424 (8)	0.0424 (8)	0.0088 (6)	−0.0039 (6)	0.0109 (6)
C11B	0.0273 (6)	0.0330 (7)	0.0450 (7)	0.0113 (5)	0.0011 (5)	0.0093 (5)
O12	0.0351 (5)	0.0309 (5)	0.0438 (5)	0.0132 (4)	0.0073 (4)	0.0085 (4)
C13	0.0395 (7)	0.0419 (7)	0.0327 (7)	0.0179 (6)	0.0056 (5)	0.0105 (5)
O13	0.0452 (6)	0.0456 (6)	0.0635 (7)	0.0234 (5)	0.0030 (5)	0.0123 (5)
O14	0.0458 (6)	0.0534 (7)	0.0541 (7)	0.0215 (5)	−0.0103 (5)	−0.0062 (5)
C14	0.0509 (11)	0.0831 (15)	0.0813 (14)	0.0250 (10)	−0.0251 (10)	−0.0073 (11)

### Geometric parameters (Å, º)

**Table:**

C1—C2	1.315 (3)	C8—C9	1.495 (4)
C1—C11B	1.5182 (19)	C8—H8A	0.9700
C1—H1	0.9300	C8—H8B	0.9700
C2—C3	1.519 (2)	C9—C10	1.484 (4)
C2—H2	0.9300	C9—H9A	0.9700
C3—O12	1.4380 (18)	C9—H9B	0.9700
C3—C4	1.567 (2)	C10—C11	1.504 (3)
C3—H3	0.9800	C10—H10A	0.9700
C4—C13	1.5062 (19)	C10—H10B	0.9700
C4—C4A	1.5450 (19)	C11—C11A	1.531 (2)
C4—H4	0.9800	C11—H11A	0.9700
C4A—C5	1.5226 (18)	C11—H11B	0.9700
C4A—C11B	1.5508 (18)	C11A—C11B	1.515 (2)
C4A—H4A	0.9800	C11A—H11C	0.9800
C5—O5	1.2228 (17)	C11B—O12	1.4386 (16)
C5—N6	1.3502 (19)	C13—O13	1.1972 (17)
N6—C7	1.454 (2)	C13—O14	1.3389 (18)
N6—C11A	1.4718 (19)	O14—C14	1.440 (2)
C7—C8	1.490 (3)	C14—H14A	0.9600
C7—H7A	0.9700	C14—H14B	0.9600
C7—H7B	0.9700	C14—H14C	0.9600
			
C2—C1—C11B	105.23 (13)	C10—C9—C8	117.5 (2)
C2—C1—H1	127.4	C10—C9—H9A	107.9
C11B—C1—H1	127.4	C8—C9—H9A	107.9
C1—C2—C3	106.25 (13)	C10—C9—H9B	107.9
C1—C2—H2	126.9	C8—C9—H9B	107.9
C3—C2—H2	126.9	H9A—C9—H9B	107.2
O12—C3—C2	100.98 (12)	C9—C10—C11	118.87 (19)
O12—C3—C4	101.71 (10)	C9—C10—H10A	107.6
C2—C3—C4	106.12 (12)	C11—C10—H10A	107.6
O12—C3—H3	115.4	C9—C10—H10B	107.6
C2—C3—H3	115.4	C11—C10—H10B	107.6
C4—C3—H3	115.4	H10A—C10—H10B	107.0
C13—C4—C4A	115.38 (11)	C10—C11—C11A	116.12 (18)
C13—C4—C3	112.85 (12)	C10—C11—H11A	108.3
C4A—C4—C3	100.04 (11)	C11A—C11—H11A	108.3
C13—C4—H4	109.4	C10—C11—H11B	108.3
C4A—C4—H4	109.4	C11A—C11—H11B	108.3
C3—C4—H4	109.4	H11A—C11—H11B	107.4
C5—C4A—C4	119.20 (10)	N6—C11A—C11B	100.81 (11)
C5—C4A—C11B	100.41 (11)	N6—C11A—C11	112.40 (13)
C4—C4A—C11B	101.76 (10)	C11B—C11A—C11	112.17 (14)
C5—C4A—H4A	111.4	N6—C11A—H11C	110.4
C4—C4A—H4A	111.4	C11B—C11A—H11C	110.4
C11B—C4A—H4A	111.4	C11—C11A—H11C	110.4
O5—C5—N6	125.20 (13)	O12—C11B—C11A	111.09 (11)
O5—C5—C4A	126.66 (13)	O12—C11B—C1	101.52 (11)
N6—C5—C4A	108.11 (11)	C11A—C11B—C1	126.86 (12)
C5—N6—C7	121.55 (14)	O12—C11B—C4A	99.30 (10)
C5—N6—C11A	114.43 (12)	C11A—C11B—C4A	105.24 (11)
C7—N6—C11A	123.77 (14)	C1—C11B—C4A	109.56 (11)
N6—C7—C8	114.54 (19)	C3—O12—C11B	96.06 (10)
N6—C7—H7A	108.6	O13—C13—O14	123.64 (13)
C8—C7—H7A	108.6	O13—C13—C4	126.23 (13)
N6—C7—H7B	108.6	O14—C13—C4	110.07 (11)
C8—C7—H7B	108.6	C13—O14—C14	115.86 (13)
H7A—C7—H7B	107.6	O14—C14—H14A	109.5
C7—C8—C9	117.0 (2)	O14—C14—H14B	109.5
C7—C8—H8A	108.0	H14A—C14—H14B	109.5
C9—C8—H8A	108.0	O14—C14—H14C	109.5
C7—C8—H8B	108.0	H14A—C14—H14C	109.5
C9—C8—H8B	108.0	H14B—C14—H14C	109.5
H8A—C8—H8B	107.3		
			
C11B—C1—C2—C3	−0.09 (17)	C10—C11—C11A—N6	65.8 (2)
C1—C2—C3—O12	−32.37 (16)	C10—C11—C11A—C11B	178.61 (16)
C1—C2—C3—C4	73.37 (16)	N6—C11A—C11B—O12	−76.52 (13)
O12—C3—C4—C13	−91.15 (13)	C11—C11A—C11B—O12	163.70 (12)
C2—C3—C4—C13	163.64 (12)	N6—C11A—C11B—C1	159.66 (13)
O12—C3—C4—C4A	32.02 (12)	C11—C11A—C11B—C1	39.9 (2)
C2—C3—C4—C4A	−73.20 (13)	N6—C11A—C11B—C4A	30.01 (13)
C13—C4—C4A—C5	17.25 (17)	C11—C11A—C11B—C4A	−89.77 (14)
C3—C4—C4A—C5	−104.12 (13)	C2—C1—C11B—O12	32.57 (15)
C13—C4—C4A—C11B	126.38 (12)	C2—C1—C11B—C11A	160.29 (14)
C3—C4—C4A—C11B	5.01 (12)	C2—C1—C11B—C4A	−71.75 (15)
C4—C4A—C5—O5	−49.29 (19)	C5—C4A—C11B—O12	82.53 (11)
C11B—C4A—C5—O5	−159.17 (14)	C4—C4A—C11B—O12	−40.49 (11)
C4—C4A—C5—N6	132.54 (12)	C5—C4A—C11B—C11A	−32.47 (12)
C11B—C4A—C5—N6	22.66 (13)	C4—C4A—C11B—C11A	−155.48 (11)
O5—C5—N6—C7	3.1 (2)	C5—C4A—C11B—C1	−171.63 (11)
C4A—C5—N6—C7	−178.69 (14)	C4—C4A—C11B—C1	65.35 (13)
O5—C5—N6—C11A	177.51 (13)	C2—C3—O12—C11B	50.39 (12)
C4A—C5—N6—C11A	−4.29 (16)	C4—C3—O12—C11B	−58.82 (11)
C5—N6—C7—C8	−152.76 (19)	C11A—C11B—O12—C3	171.91 (11)
C11A—N6—C7—C8	33.4 (3)	C1—C11B—O12—C3	−50.81 (12)
N6—C7—C8—C9	43.2 (3)	C4A—C11B—O12—C3	61.51 (11)
C7—C8—C9—C10	−81.4 (3)	C4A—C4—C13—O13	−91.08 (18)
C8—C9—C10—C11	62.4 (3)	C3—C4—C13—O13	23.1 (2)
C9—C10—C11—C11A	−48.4 (3)	C4A—C4—C13—O14	91.77 (14)
C5—N6—C11A—C11B	−16.60 (16)	C3—C4—C13—O14	−154.06 (12)
C7—N6—C11A—C11B	157.66 (15)	O13—C13—O14—C14	1.6 (2)
C5—N6—C11A—C11	103.02 (16)	C4—C13—O14—C14	178.86 (16)
C7—N6—C11A—C11	−82.7 (2)		

### Hydrogen-bond geometry (Å, º)

**Table:**

*D*—H···*A*	*D*—H	H···*A*	*D*···*A*	*D*—H···*A*
C1—H1···O5^i^	0.93	2.59	3.4576 (19)	156
C3—H3···O13^ii^	0.98	2.55	3.5259 (19)	174
C4*A*—H4*A*···O14^iii^	0.98	2.51	3.4190 (17)	154
C14—H14*A*···O5^iv^	0.96	2.56	3.279 (2)	132

Symmetry codes: (i) *x*−1, *y*, *z*; (ii) −*x*+2, −*y*, −*z*+2; (iii) −*x*+2, −*y*+1, −*z*+2; (iv) −*x*+3, −*y*+1, −*z*+2.
