# Supplementary figures and images for: Crystal structure of methyl (3RS,4SR,4aRS,11aRS,11bSR)-5-oxo-3,4,4a,5,7,8,9,10,11,11a-deca­hydro-3,11b-ep­oxy­azepino[2,1-a]iso­indole-4-carboxyl­ate
Source: Acta Crystallogr E Crystallogr Commun. 2015 Sep 12;71(Pt 10):o729–30. doi: 10.1107/S2056989015016679 (PMC4647423; doi:10.1107/S2056989015016679)

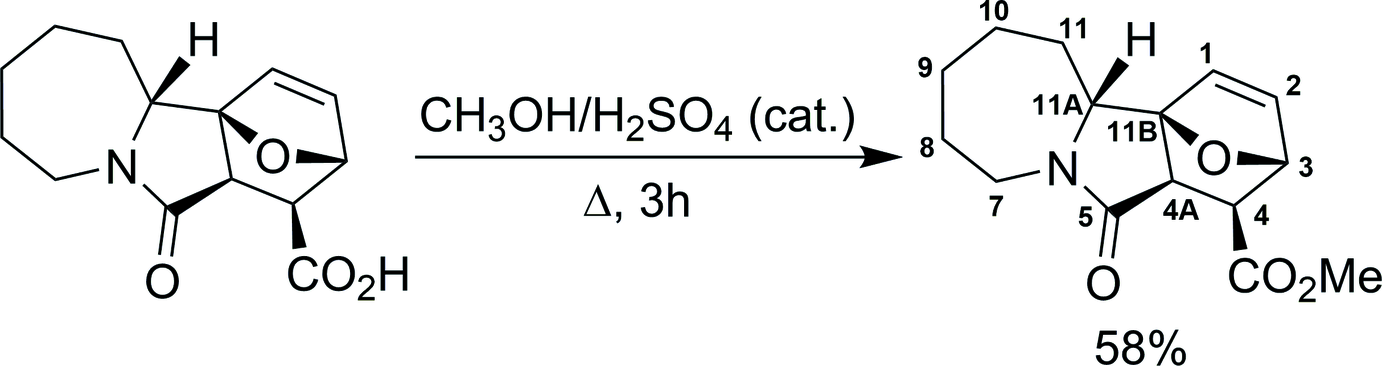

Supplement: Supplementary file 4 [file e-71-0o729-fig1.tif]

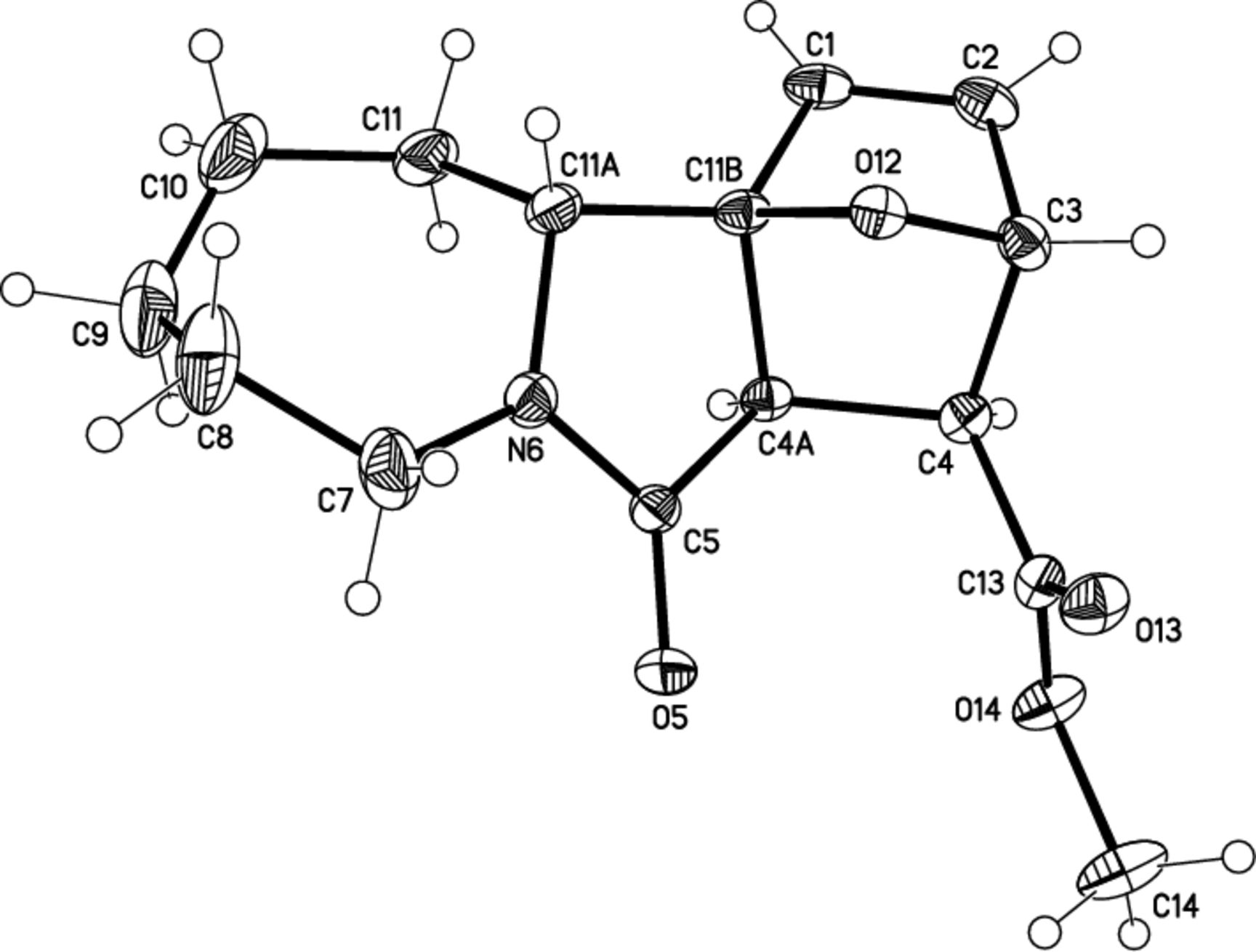

Supplement: Supplementary file 5 [file e-71-0o729-fig2.tif]

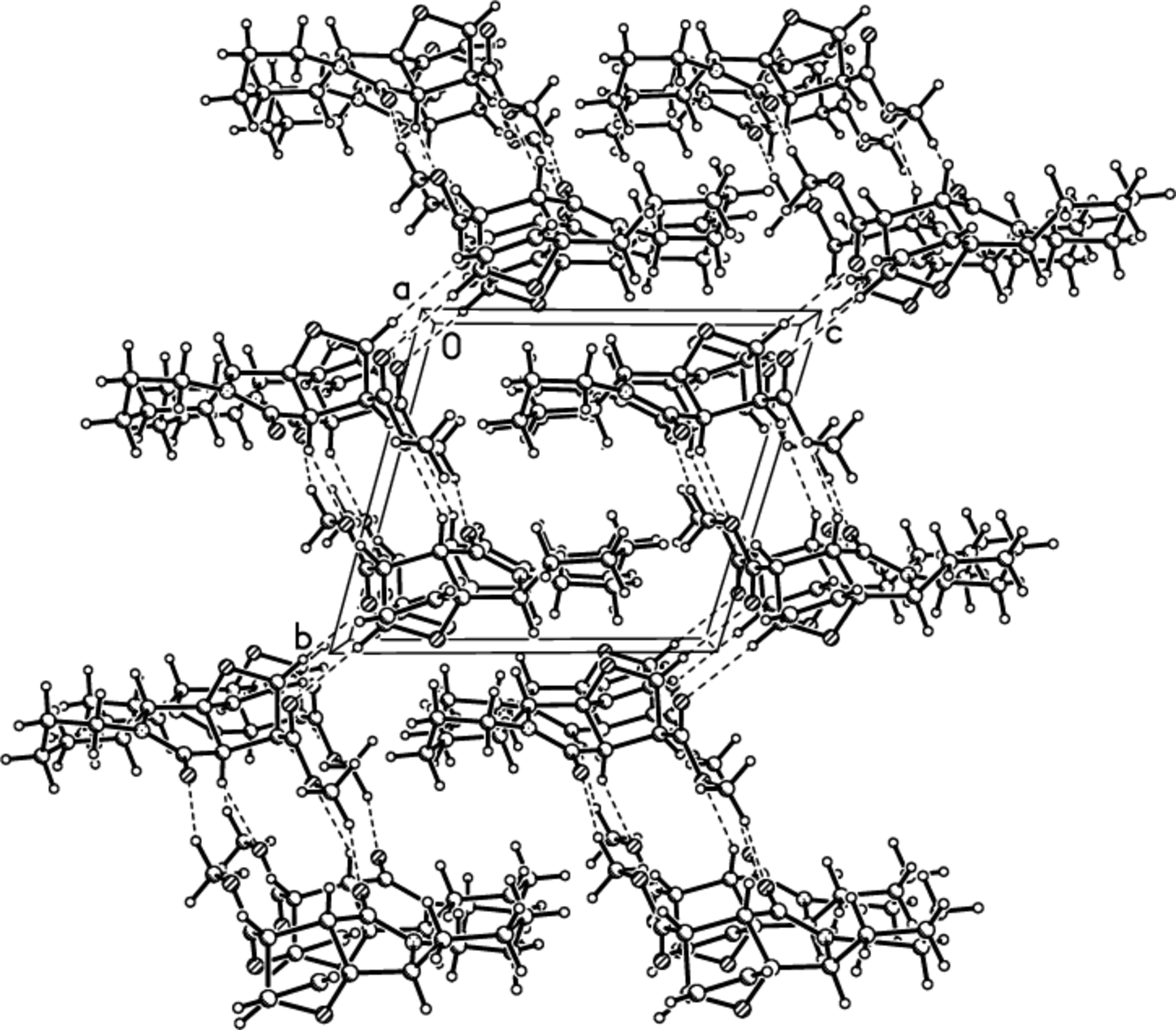

Supplement: Supplementary file 6 [file e-71-0o729-fig3.tif]
